# Sirtuin3 protects aged human mesenchymal stem cells against oxidative stress and enhances efficacy of cell therapy for ischaemic heart diseases

**DOI:** 10.1111/jcmm.13821

**Published:** 2018-08-09

**Authors:** Dong‐Yang Zhang, Chun‐Feng Zhang, Bi‐Cheng Fu, Lu Sun, Xue‐Qing Wang, Wei Chen, Wei Liu, Kai‐Yu Liu, Guo‐Qing Du, Chong‐Yi Ma, Shu‐Lin Jiang, Ren‐Ke Li, Hai Tian

**Affiliations:** ^1^ Department of Cardiovascular Surgery Second Affiliated Hospital of Harbin Medical University Harbin Heilongjiang China; ^2^ Key Laboratory of Myocardial Ischemia Harbin Medical University Ministry of Education Harbin Heilongjiang China; ^3^ Toronto General Hospital Research Institute University Health Network Toronto Ontario Canada; ^4^ Department of Surgery University of Toronto Toronto Ontario Canada

**Keywords:** ageing, antioxidant capacity, cell transplantation, gene modification, human mesenchymal stem cells

## Abstract

Sirtuin3 (SIRT3) is associated with oxidative stress and lifespan. However, the possible mechanisms underlying its influence are unknown. We hypothesized that SIRT3 increases the antioxidant capacity of aged cells and improves the efficacy of human mesenchymal stem cell (hMSC) therapy for ischaemic heart diseases in aged patients. In vitro, the antioxidant capacity of old hMSCs (O‐hMSCs) was increased after SIRT3 overexpression using a gene transfection technique, while the antioxidant capacity of young hMSCs (Y‐hMSCs) was decreased by SIRT3 silencing. The levels of forkhead box O3a (FoxO3a) in the nucleus, and antioxidant enzymes Mn‐superoxide dismutase (MnSOD) and catalase (CAT) increased in SIRT3‐overexpressed O‐hMSCs while they decreased in SIRT3‐silenced Y‐hMSCs after oxidative stress. Following myocardial infarction in adult rats in vivo, infarct size decreased and cardiac function was significantly enhanced after cell transplantation with SIRT3 overexpressed O‐hMSCs. The number of apoptotic cells decreased and the survival rate of transplanted cells increased following SIRT3 overexpression in O‐hMSCs. SIRT3 protects aged hMSCs against oxidative stress by positively regulating antioxidant enzymes (MnSOD and CAT) via increasing the expression of FoxO3a in the nucleus. The efficacy of aged hMSC transplantation therapy for ischaemic heart diseases can be improved by SIRT3 overexpression.

## 1 INTRODUCTION

As the size of the ageing population continues to increase, age‐related ischaemic heart diseases have become a primary contributor to mortality.^1^ Current treatments such as percutaneous coronary intervention or coronary artery bypass grafting have improved patients’ quality of life; however, long‐term cardiac function and survival in aged patients remain poor^2^, ^3^.

Cell transplantation, especially mesenchymal stem cell (MSC) transplantation, has been extensively studied in recent years as a therapy for ischaemic heart diseases.^4^, ^5^, ^6^ In early animal experiments, the treatment effect of young stem cell transplantation was satisfactory.^5^ However, the therapeutic effect from old autologous stem cell transplantation was found to be limited in clinical trials.^7^, ^8^ Our previous studies have revealed the close association between stem cell function and ageing.^9^, ^10^ The regenerative capacity, biological activity and resistance to oxidative stress of stem cells significantly decline with age. This suggests that stem cell senescence is likely to be a major contributing factor to the ineffectual cell transplantation outcomes found in the clinical setting. We hypothesized that enhancing the function of old stem cells may improve the therapeutic effect of transplantation.

Sirtuin3 (SIRT3) is a deacetylase from the sirtuin family.^11^, ^12^ There is significant evidence that SIRT3 localizes to mitochondria and plays a positive role in human longevity, organ ageing and stem cell function.^13^, ^14^, ^15^ Human SIRT3 has two isoforms: a full‐length isoform (fl‐SIRT3) and a short one (sh‐SIRT3) which is considered to have the most functional significance.^16^, ^17^, ^18^, ^19^ Recent studies have reported that SIRT3 reduces levels of reactive oxygen species (ROS) by deacetylating the transcription factor forkhead box O3a (FoxO3a), which can then enter the nucleus and bind to the promoter of the genes encoding Mn‐superoxide dismutase (MnSOD) and CAT.^20^, ^21^, ^22^ Furthermore, many researchers have demonstrated that the elevated expression of MnSOD and CAT by SIRT3 overexpression can protect cells against the oxidative damage induced by ROS.^10^, ^17^

In our previous study, we determined that the lower expression of sh‐SIRT3 in old relative to young human myocardial tissue significantly contributed to myocardial ageing.^10^ In addition, we showed that the decline in antioxidant capacity of old human MSCs (O‐hMSCs) is because of a substantially greater down‐regulation of SIRT3 than that which occurs in young hMSCs (Y‐hMSCs) under oxidative stress.^10^ However, there is little evidence to support the potential association between SIRT3 and O‐hMSC function. In this study, we explored the relationship and underlying mechanisms involved in SIRT3's influence on the antioxidant capacity of O‐hMSCs. We further investigated the efficacy of cell transplantation therapy when SIRT3‐modified O‐hMSCs were injected into the infarct region of the heart using a rat myocardial infarction (MI) model.

## 2 MATERIALS AND METHODS

### 2.1 Isolation and culture of hMSCs

Human bone marrow was acquired from the sternum of patients who underwent cardiac surgery at the Second Affiliated Hospital of Harbin Medical University. Informed consent was obtained for study participation, and the rights of infants and young children were entrusted to their parents. All protocols were reviewed and approved by the Research Ethics Board of the Harbin Medical University and conformed to the principles of the Declaration of Helsinki. O‐hMSCs were obtained from patients 50‐72 years of age (mean = 61.3 ± 9.5), and Y‐hMSCs were obtained from patients 0‐12 years of age (mean = 5.0 ± 4.9). MSCs were isolated by density gradient separation with Ficoll‐Paque premium (1.073 g/mL density; GE Healthcare, Uppsala, Sweden) as described previously.^10^ The mononuclear cells were plated into 25 cm^2^ culture flasks (Corning, Corning, NY, USA) in Dulbecco's modified Eagle's medium (DMEM, low glucose; Gibco, Grand Island, NY, USA) supplemented with 10% foetal bovine serum (FBS; Sciencell, San Diego, CA, USA). Finally, the cells were incubated at 37°C with 5% CO_2_. Third‐generation hMSCs were harvested for the following experiments.

### 2.2 Plasmid and siRNA constructs

A plasmid containing a SIRT3 expression gene (pSIRT3) was constructed on a pEX‐1 (pGCMV/MCS/EGFP/NEO) backbone (GenePharma, Shanghai, China). The plasmids were amplified and prepared using the Endofree maxi plasmid kit (TIANGEN, Beijing, China). The negative control (NC) and small interfering RNA (siRNA) were designed and synthesized by GenePharma to interfere with SIRT3 expression.

### 2.3 Plasmid and siRNA transfection

The plasmid transfection was performed in accordance with the manufacturer's instructions (X‐tremeGENE HP DNA transfection reagent; Roche, Penzberg, Upper Bavaria, Germany). After 72‐hour culture, transfection efficiency was assayed by fluorescence activated cell sorting (FACS; BD, Franklin Lakes, NJ, USA). The siRNA transfection was performed in accordance with the manufacturer's instructions (X‐tremeGENE HP siRNA transfection reagent; Roche).

### 2.4 Cell stress experiment

The transfection was performed as described above. Seventy‐two hours after transfection, experimental group cells were treated with hydrogen peroxide (H_2_O_2_) diluted with DMEM [800 μmol/L for O‐hMSCs; 1 mmol/L for Y‐hMSCs] (Sigma‐Aldrich, St. Louis, MO, USA) for 1 hour. Control groups were treated by DMEM (without H_2_O_2_) for 1 hour. All cells were then harvested for the following experiments. hMSCs were divided into eight groups, with four groups using O‐hMSCs and four using Y‐hMSCs. For O‐hMSCs, the groups were pEX‐1 transfection group without H_2_O_2_ treatment (Control), pSIRT3 transfection group without H_2_O_2_ treatment (SIRT3), pEX‐1 transfection + H_2_O_2_ treatment group (Control+) and pSIRT3 transfection + H_2_O_2_ treatment group (SIRT3+). For Y‐hMSCs, the groups were negative control transfection group without H_2_O_2_ treatment (Control), SIRT3 siRNA transfection group without H_2_O_2_ treatment (siSIRT3), NC transfection + H_2_O_2_ treatment group (Control+) and SIRT3 siRNA transfection + H_2_O_2_ treatment group (siSIRT3+).

### 2.5 Cell apoptosis and survival evaluation in vitro

Cell apoptosis was assessed by terminal dUTP nick‐end labelling (TUNEL) with an In Situ Cell Death Detection Kit, TMR red (Roche, Indianapolis, IN, USA) according to the manufacturer's protocol after oxidative stress. The number of apoptotic cells was determined by counting the cells of TUNEL‐positive nuclei per microscopic field (200X = 0.4 mm^2^) in five fields per sample and then averaging the results. Cell Counting Kit‐8 (CCK8; Dojindo, Kumamoto, Japan) was used to determine cell survival according to the manufacturer's instructions after oxidative stress.

### 2.6 Gene expression measurement

Total RNA was extracted directly from hMSCs using TRIzol reagent (Life Technologies, Carlsbad, CA, USA) after oxidative stress. RNA samples were treated with DNase I (Sigma‐Aldrich) according to the manufacturer's protocol. Reverse transcription was performed with AccuPower RocketScript RT PreMix (Bioneer, Alameda, CA, USA) according to the manufacturer's protocol. The primers were designed and synthesized by Bioneer Corporation as shown in Table 1. The gene expression level of SIRT3 was determined by real‐time PCR with AccuPower 2×GreenstarqPCR Master Mix (Bioneer) on a thermal cycler (Bio‐Rad, Hercules, CA, USA) at an annealing temperature of 56°C for 40 cycles (each cycle: 95°C for 10 seconds, 56°C for 30 seconds). In addition, gene expression levels of MnSOD and CAT were measured at an annealing temperature of 54.4°C for 40 cycles. Relative gene expression was calculated by comparing the Δ*C*_t_ values between control and experimental conditions for each PCR target using the following equation: relative gene expression = $2^{-(\Delta C_{t}\mathrm{sample}-\Delta C_{t}\mathrm{control})}$.

**Table 1:** Primers used for quantitative real‐time PCR

Name	Sequence
β‐actin‐F	5′‐CCCAGCACAATGAAGATCAAGATCAT‐3′
β‐actin‐R	5′‐ATCTGCTGGAAGGTGTACAGCGA‐3′
SIRT3‐F	5′‐GCATCCCTGCCTCAAAGC‐3′
SIRT3‐R	5′‐CGTCAGCCCGAATGTCCTC‐3′
MnSOD‐F	5′‐TGGTGGTCATATCAATCATAGC‐3′
MnSOD‐R	5′‐ATTTGTAAGTGTCCCCGTTC‐3′
CAT‐F	5′‐TGCTGAGGTTGAACAGATAG‐3′
CAT‐R	5′‐CCGTCACGCTGGTAGTT‐3′

CAT: catalase; MnSOD: Mn‐superoxide dismutase; SIRT3: Sirtuin3.

### 2.7 Protein level measurement

Protein expression levels of SIRT3, MnSOD, CAT and FoxO3a were determined by Western blot. Total protein was extracted from cells using RIPA lysis buffer (Millipore, Temecula, CA, USA) using a protease inhibitor cocktail tablet (Complete, ULTRA, Mini, EDTA‐free, Easypack; Roche) followed by oxidative stress, while nuclear protein was extracted using Nuclear Protein Extraction Kit (Solarbio, Beijing, China). Protein concentration of all samples was measured with a BCA Protein Assay Kit (Beyotime, Haimen, Jiangsu, China) according to the manufacturer's protocol on a microplate reader (TECAN, Mannedorf, Switzerland). Equal amounts of protein were applied to 12% acrylamide gels and electrophoresed. Then, the samples were electroblotted onto polyvinylidene difluoride (PVDF, Roche) membranes and probed with antibodies. Antibodies used were as follows: rabbit monoclonal antibody against sh‐SIRT3(C73E3; 1:1000; Cell Signaling technology, Danvers, MA, USA), rabbit polyclonal antibody against fl‐SIRT3 (1:1000; Abgent, San Diego, CA, USA), rabbit monoclonal antibody against SOD2 (1:1000, MnSOD; OriGene, Beijing, China), rabbit monoclonal antibody against catalase (1:10000, CAT; Abcam, Cambridge, UK), rabbit monoclonal antibody against FoxO3a (1:2500; Abcam), rabbit polyclonal antibody against Histone H3 (1:2500; Abcam), mouse anti‐β‐actin (1:1000; ZSGB‐BIO, Beijing, China), goat anti‐rabbit immunoglobulin G (IgG)‐horseradish peroxidase (HRP; 1:8000; ZSGB‐BIO) and goat antimouse IgG‐HRP (1:8000; ZSGB‐BIO). Specific complexes were visualized on an X‐ray film using electrochemi‐luminescence (ECL) detection (Millipore) following the manufacturer's protocol. The levels of proteins were compared among groups by density and area using Quantity One software (Bio‐Rad).

### 2.8 Enzyme activity measurement of MnSOD and CAT

To measure enzyme activity, cultured cells were collected and protein extracted as outlined above. Enzyme activity was detected using the MnSOD Assay and CAT Assay kits (Beyotime) according to the manufacturer's protocol.

### 2.9 Animal model and cell transplantation

All experiments were carried out according to the Guide for the Care and Use of Laboratory Animals (NIH, 8th Edition, 2011), and research protocols were approved by Animal Care Committee of Harbin Medical University. Adult male Sprague‐Dawley (SD) rats (210‐230 g) were obtained from the Central Animal Laboratory of the Second Affiliated Hospital of Harbin Medical University, China. All rats were immunosuppressed with intraperitoneal injections of cyclosporine A (5 mg/kg; Novartis, Basel, Switzerland) each day from 3 days before cell transplantation until the end of the experiment. Myocardial infarction (MI) was performed. Briefly, rats were deeply anaesthetized and respiration was assisted with endotracheal intubation (arterial puncture needle, 16G) via a ventilator (Harvard Inspira ASVp; NatureGene Corp., Medford, NJ, USA) with 2% isoflurane and buprenorphine (0.05 mg/kg) given for analgesia. Through a left lateral thoracotomy, an MI was created by ligating the proximal left coronary artery (LAD) with 5‐0 Prolene. MI was confirmed by an immediate change in regional colour (blanching). Fifteen minutes after MI, cells suspended in DMEM (2 × 10^6^/100 μL) were injected into one site in the centre of the ischaemic myocardium and four sites in the border of the infarct region. The incision was closed. Rats were divided into three groups: transplantation with DMEM only group (Control), transplantation using O‐hMSCs transfected with pEX‐1 group (O‐hMSCs‐pEX‐1) and transplantation using O‐hMSCs transfected with pSIRT3 group (O‐hMSCs‐pSIRT3).

### 2.10 Infarct size measurement

Four weeks after MI and cell transplantation, rats were anaesthetized and respiration was assisted as described above. The heart was exposed through a median sternotomy, and 0.9% normal saline was continuously injected into the left ventricle of rats through the apex cordis using a syringe (20 mL). Meanwhile, the right atrium was carved to reduce the circulation load. Cardiac arrest in diastole was performed by injecting 10% KCL into the left ventricle when the myocardium blanched. Hearts were then excised, and an intraventricular balloon was inserted through the mitral valve and filled to 20 mm Hg pressure. Hearts were then fixed in formalin for 1 week under controlled pressure conditions and sliced. Sections were stained with Masson's trichrome to assess infarct size.

### 2.11 Cardiac function assessment

Prior to and 1, 2 and 4 weeks after MI, rats were anaesthetized as above and fixed in the right lateral decubitus position. Echocardiography was performed with a 12‐MHz transducer (Vivid 7; GE Healthcare), and left parasternal images were obtained. The left ventricular end diastolic volume (LVEDV) and the left ventricular end systolic volume (LVESV) were measured by two‐dimensional and M‐mode images. Left ventricular ejection fraction (EF) and fractional shortening (FS) were calculated automatically by the echocardiography system. All echocardiography data were averaged from at least three consecutive cardiac cycles.

### 2.12 Cell apoptosis and survival evaluation in vivo

Three days after MI and cell transplantation, rats were sacrificed. Briefly, rats were anaesthetized and respiration was assisted as above. The heart was exposed through a median sternotomy, the right atrium was carved to reduce the circulation load and 0.9% normal saline was continuously injected into the left ventricle through the apex cordis using a syringe (20 mL) until cardiac arrest occurred. The hearts were excised as described above and sliced into 2‐mm thick sections and fixed with 4% paraformaldehyde for 24 hours, followed by sucrose at varying concentrations and durations (10% for 1 hour, 20% for 1 hour and 30% for 24 hours). The tissues were then snap‐frozen in moulds filled with optimal cutting temperature (OCT) compound (SAKURA, Torrance, CA, USA). The OCT‐embedded tissues were cut into 5‐μm‐thick sections using a freezing microtome. Cell apoptosis was assessed by TUNEL according to the manufacturer's protocol. The number of apoptotic cells was determined by counting the number of TUNEL‐positive nuclei per microscopic field (400X = 0.2 mm^2^) in five fields per sample and then averaging the results.

The hearts were excised, sliced, fixed, dehydrated, embedded and cut 3, 7 and 28 days after cell transplantation as described above. The frozen sections were incubated with human‐specific anti‐mitochondria antibody (1:48; Millipore) and goat antimouse IgG‐Texas Red (1:200; Santa Cruz, Dallas, TX, USA) according to the manufacturer's protocol. Then, the sections were immersed in 4′,6‐diamidino‐2‐phenylindole (DAPI; Sigma‐Aldrich) for 5 minutes to stain nuclei. The number of remaining cells was determined by counting positive cells (blue nuclei surrounded by red cytoplasm) using immunofluorescence per microscopic field (400X = 0.2 mm^2^) in five fields per slide and then averaging the results.

### 2.13 Statistical analysis

Data are expressed as mean ± standard deviation (SD). Analyses were conducted using GraphPad Prism 6.0 software (GraphPad, La Jolla, CA, USA). Comparisons between two groups were performed using two‐tailed Student's *t* test. One‐way ANOVA was used to determine the significance between three or more experimental groups. Repeated‐measures ANOVA was used for left ventricular systolic function (ejection fraction and fractional shortening). *P* < 0.05 was considered statistically significant.

## 3 RESULTS

### 3.1 Expression of SIRT3, MnSOD and CAT increase after transfection of SIRT3 in O‐hMSCs

**Figure 1:**
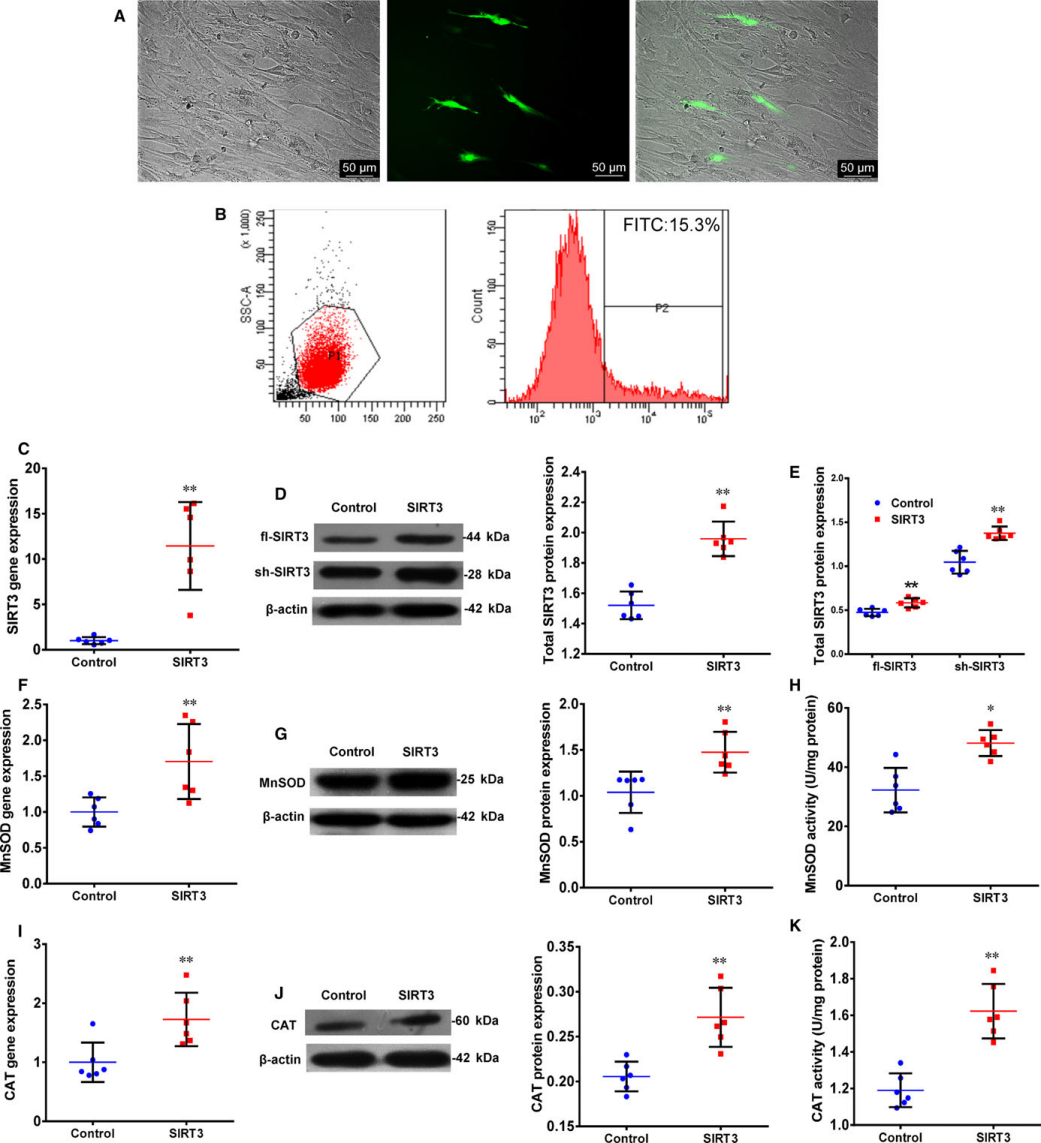
Transfection of sirtuin3 (SIRT3) enhanced expression of SIRT3, Mn‐superoxide dismutase (MnSOD) and catalase (CAT) in old human mesenchymal stem cells (hMSCs). A,B, Transfection efficiency of O‐hMSCs (14.1 ± 1.7%) was detected by fluorescence activated cell sorting (FACS). Scale bars represent 50 μm. C,D, Gene and total protein expression of SIRT3 was significantly higher in the SIRT3 group than the Control group. E, Protein expression level of fl‐SIRT3 and sh‐SIRT3 was significantly greater in the SIRT3 group compared with the Control group. F,G,I,J, Gene and protein expression of MnSOD and CAT was significantly greater in the SIRT3 group compared with the Control group. H,K, Enzyme activity of MnSOD and CAT was significantly greater in the SIRT3 group compared with the Control group (two‐tailed *t* test; *P < 0.05; **P < 0.01; n = 6/group)

To study the effect of SIRT3 on the function of O‐hMSCs, SIRT3 was transfected into O‐hMSCs. Flow cytometry data showed that transfection efficiency was 14.1 ± 1.7% (Figure 1A,B, n = 6/group). The gene and total protein expression of SIRT3 in the SIRT3 group was significantly higher compared to the Control group (Figure 1C,D, n = 6/group). Moreover, the protein expression of fl‐SIRT3 and sh‐SIRT3 both increased in the SIRT3 group compared with the Control group (Figure 1E, n = 6/group). To further verify the activity of SIRT3 after transfection, gene and protein expression as well as activity of MnSOD and CAT were found to be increased after SIRT3 overexpression (Figure 1F‐K, n = 6/group). These results demonstrate that SIRT3 was successfully overexpressed in O‐hMSCs using plasmid transfection, which correlated with antioxidant activity.

### 3.2 Enhanced antioxidant capacity in O‐hMSCs protects cells from oxidative stress injury

**Figure 2:**
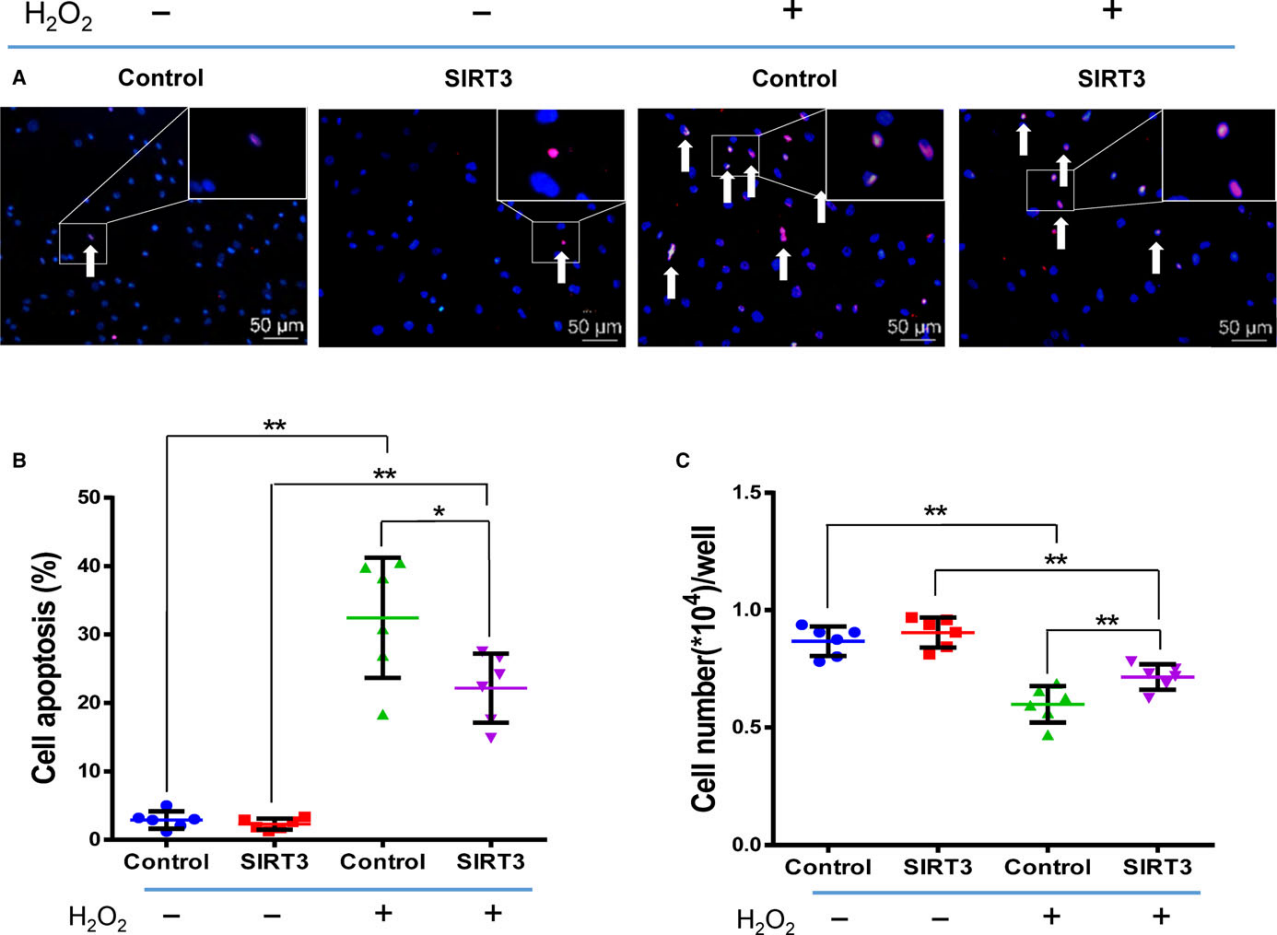
Sirtuin3 (SIRT3) overexpression increased antioxidant capacity of old human mesenchymal stem cells (hMSCs). A, Terminal dUTP nick‐end labelling (TUNEL) staining of apoptotic cells (arrows) in the four groups. Scale bars represent 50 μm. B, The cell apoptosis rate in the SIRT3+ group was significantly lower than the Control+ group, while there was no significant difference between the SIRT3 and Control group. C, The number of cells that survived was significantly higher in the SIRT3+ group than the Control+ group, and there was no significant difference between the SIRT3 and Control groups. (ANOVA, *P < 0.05; **P < 0.01; n = 6/group)

**Figure 3:**
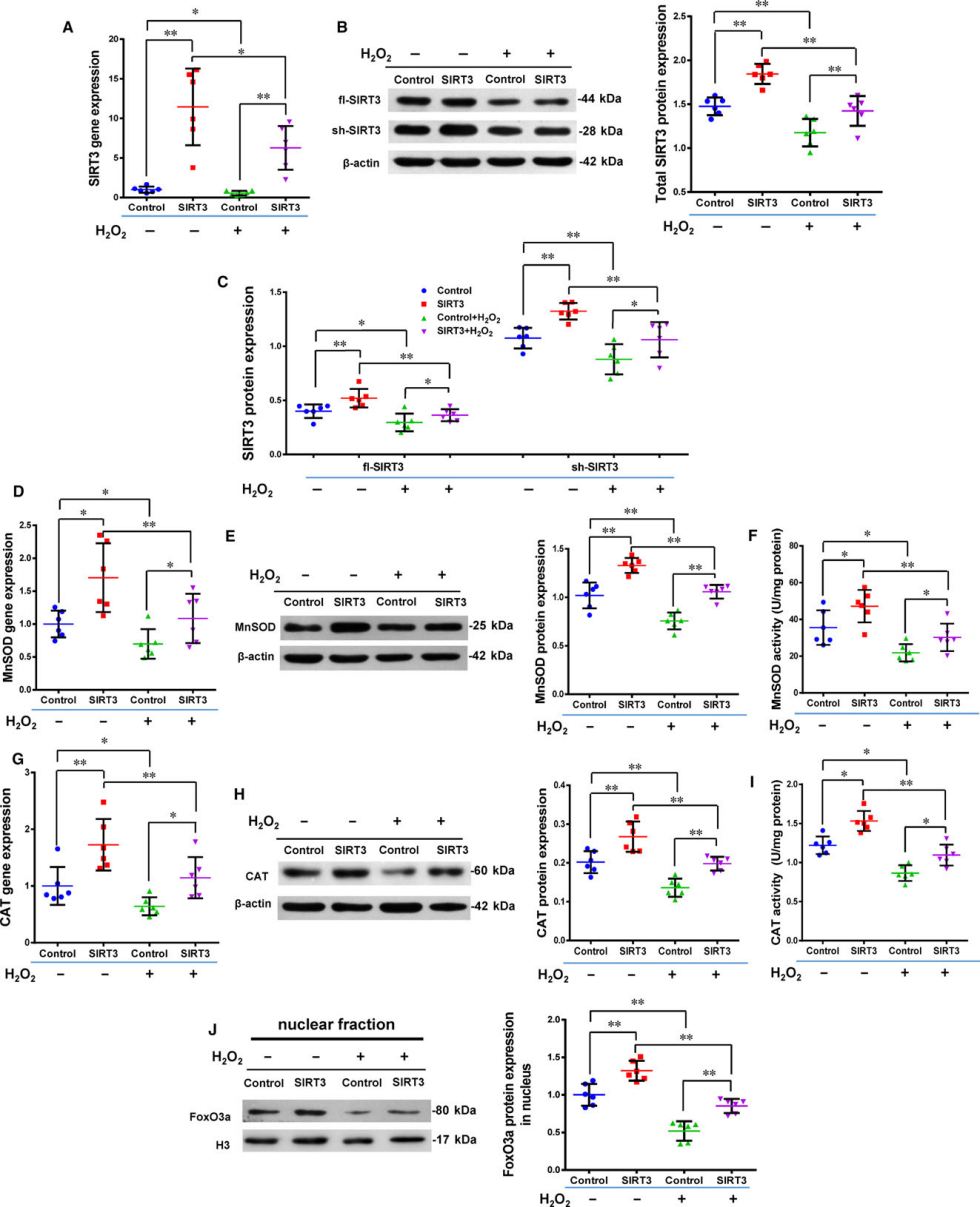
Sirtuin3 (SIRT3) overexpression increased Mn‐superoxide dismutase (MnSOD) and catalase (CAT) levels via increasing the expression of forkhead box O3a (FoxO3a) in the nucleus. A,B, The gene and total protein expression of SIRT3 was significantly higher in the SIRT3 group than the Control group with and without H_2_O_2_ treatment. C, The protein level of fl‐SIRT3 and sh‐SIRT3 was significantly higher in the SIRT3 group than the Control group with and without H_2_O_2_ treatment. D,E,H,,I, The gene and protein level of MnSOD and CAT was significantly higher in the SIRT3 than the Control group with and without H_2_O_2_ treatment. F,G, The enzyme activity of MnSOD and CAT was significantly higher in the SIRT3 than the Control group with and without H_2_O_2_ treatment. J, The protein level of FoxO3a in the nucleus was significantly higher in the SIRT3 group than the Control group with and without H_2_O_2_ treatment. (ANOVA; *P < 0.05; **P < 0.01; n = 6/group)

As shown in Figure 2, the rate of apoptosis increased while the rate of cell survival decreased after oxidative stress induced by H_2_O_2_. The apoptosis rate was significantly lower and the survival rate significantly higher in the SIRT3+ group than the Control+ group, while there were no significant differences between the SIRT3 and Control groups, which were free from oxidative stress (n = 6/group). We further measured the expression of total SIRT3 (t‐SIRT3), fl‐SIRT3, sh‐SIRT3, MnSOD, CAT and FoxO3a to explore underlying mechanisms (Figure 3, n = 6/group). After H_2_O_2_ treatment, the expression of t‐SIRT3, fl‐SIRT3 and sh‐SIRT3 was significantly lower in the Control+ and SIRT3+ groups than in the non‐H_2_O_2_‐treated groups (Control and SIRT3). However, these levels were still significantly higher in the SIRT3+ group than the Control+ group (Figure 3A‐C). The mRNA, protein and enzyme activity levels of MnSOD and CAT which decreased after oxidative stress were still significantly higher in the SIRT3+ group than the Control+ group (Figure 3D‐I).

After pSIRT3 transfection, FoxO3a protein expression significantly increased in the nuclear fraction (Figure 3J). After H_2_O_2_ treatment, the expression of FoxO3a protein in the nucleus was significantly lower in the Control+ and SIRT3+ groups than the Control and SIRT3 groups. However, the level was still significantly higher in the SIRT3+ group than the Control+ group (Figure 3J). These findings suggest that the antioxidant capacity of O‐hMSCs was enhanced by SIRT3 overexpression, likely by increasing expression and activity of MnSOD and CAT via transferring FoxO3a into the nucleus.

### 3.3 Silencing SIRT3 reduces antioxidant capacity of Y‐hMSCs

**Figure 4:**
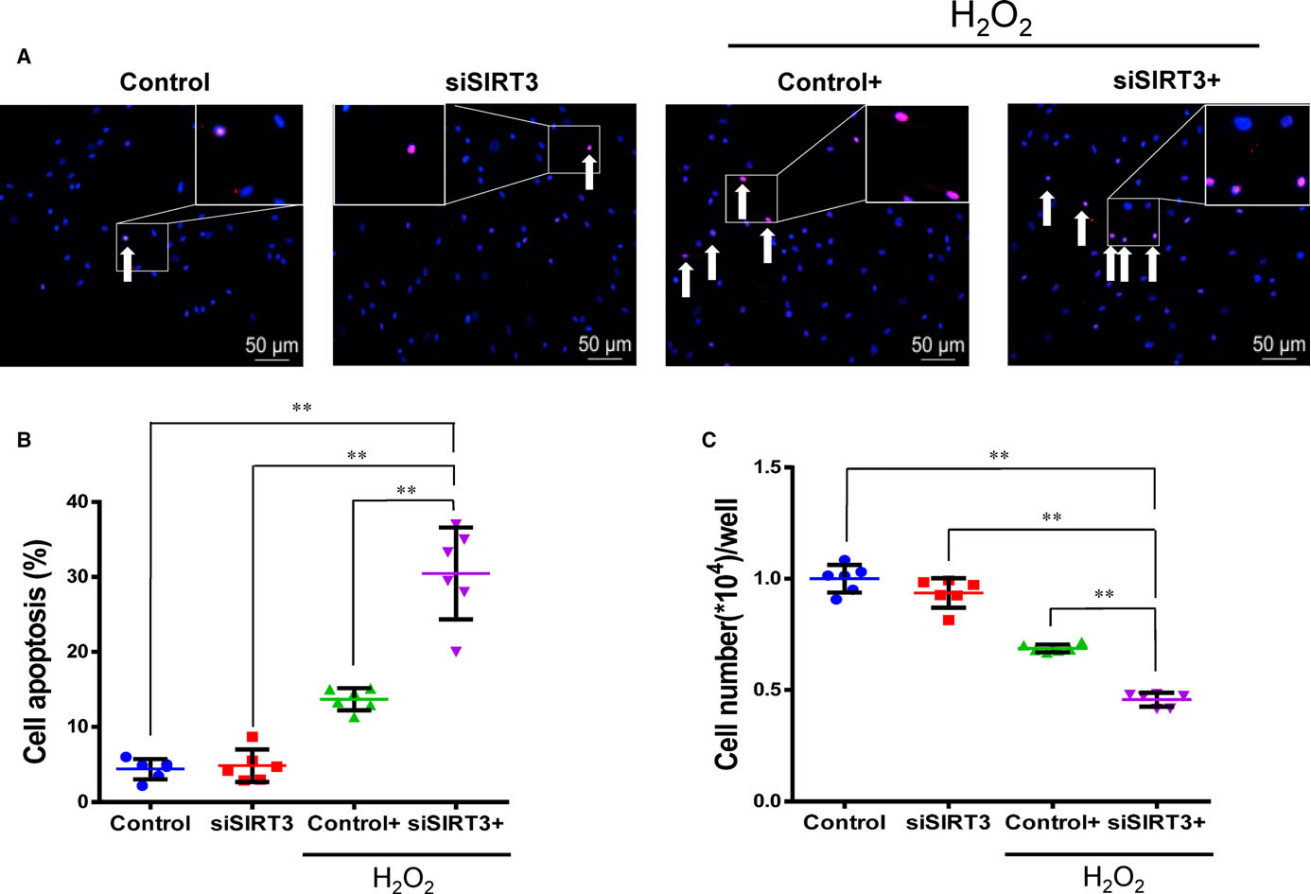
Sirtuin3 (SIRT3) silencing weakened the antioxidant capacity of young human mesenchymal stem cells (hMSCs) against oxidative stress. A, Terminal dUTP nick‐end labelling (TUNEL) staining of apoptotic cells (arrows) in the four groups. Scale bars represent 50 μm. B, The rate of cell apoptosis was significantly higher in the siSIRT3+ than the Control+ group while there was no significant difference between the siSIRT3 and Control groups. C, The cell survival rate in the siSIRT3+ group was significantly lower than the Control+ group, and there was no significant difference between the siSIRT3 and Control groups. (ANOVA; **P < 0.01; n = 6/group)

**Figure 5:**
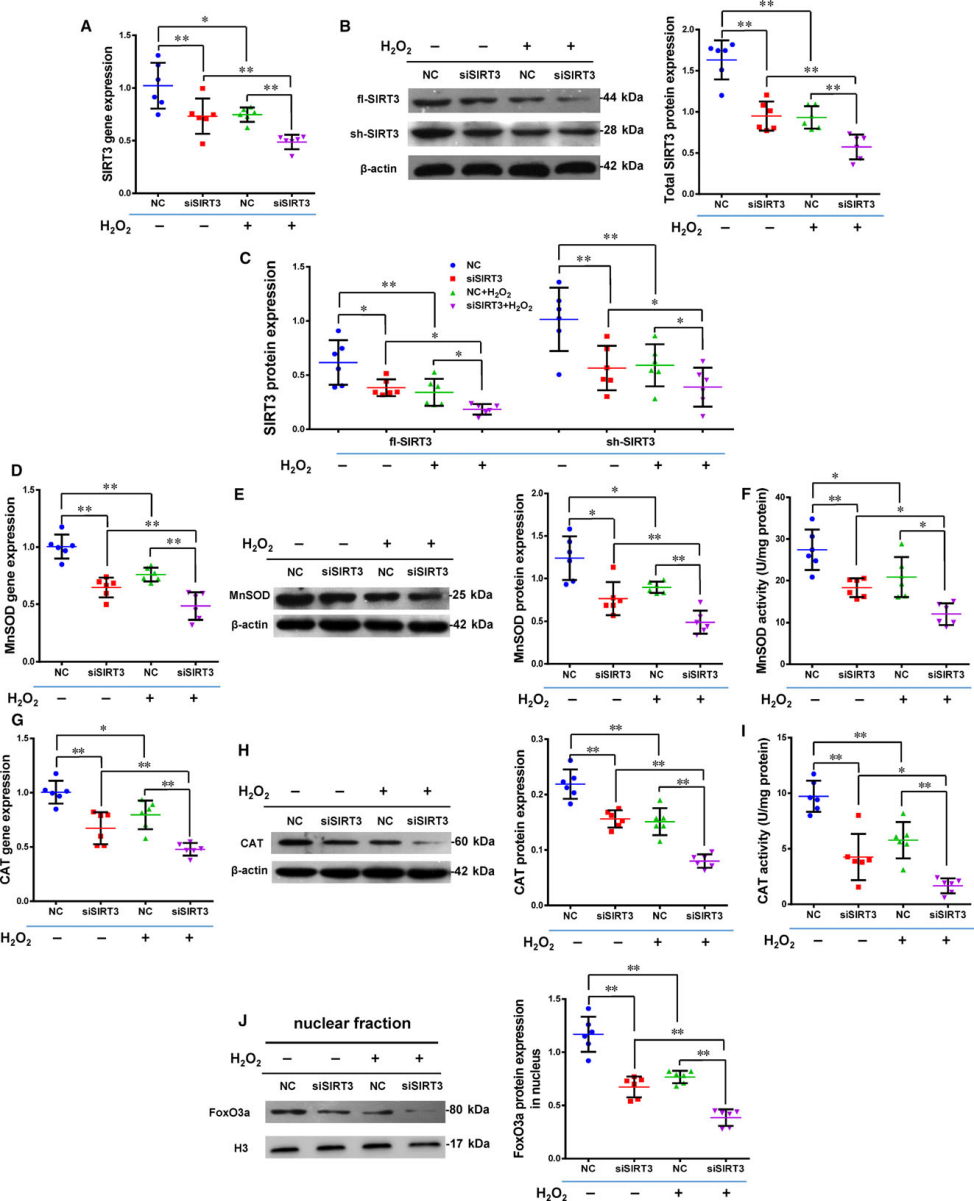
Sirtuin3 (SIRT3) silencing reduced Mn‐superoxide dismutase (MnSOD) and catalase (CAT) levels via decreasing the expression of forkhead box O3a (FoxO3a) in the nucleus. A,B, The gene and total protein expression of SIRT3 was significantly lower in the siSIRT3 than Control groups with and without H_2_O_2_ treatment. C, The protein level of fl‐SIRT3 and sh‐SIRT3 was significantly lower in the siSIRT3 than the Control group with and without H_2_O_2_ treatment. D,E,G,H, The gene and protein level of MnSOD and CAT was significantly lower in the siSIRT3 than the Control group with and without H_2_O_2_ treatment. F,I, The enzyme activity of MnSOD and CAT was significantly lower in the siSIRT3 than the Control group with and without H_2_O_2_ treatment. J, The protein level of FoxO3a in the nucleus was significantly lower in the siSIRT3 than Control group with and without H_2_O_2_ treatment. (ANOVA; *P < 0.05; **P < 0.01; n = 6/group)

The expression of SIRT3 was reduced using silencing RNA transfection in Y‐hMSCs. The rate of apoptosis increased while the cell survival rate decreased after cells were exposed to H_2_O_2_ treatment. Cellular apoptosis was significantly higher and cell survival significantly lower in the siSIRT3+ group than the Control+ group, and there were no differences between the Control and siSIRT3 groups (without H_2_O_2_ treatment; Figure 4A‐C, n = 6/group). Levels of t‐SIRT3, fl‐SIRT3, sh‐SIRT3, MnSOD and CAT were down‐regulated by H_2_O_2_ treatment in both the siSIRT3 and Control groups, but all levels were significantly lower in the siSIRT3+ group than the Control+ group (Figure 5A‐I, n = 6/group).

After siRNA transfection, FoxO3a protein expression significantly decreased in the nuclear fraction (Figure 5J, n = 6/group). The level of FoxO3a in the nuclear fraction was down‐regulated by H_2_O_2_ treatment in both the siSIRT3 and Control groups, but this level was still significantly lower in the siSIRT3+ group than the Control+ group (Figure 5J). These results suggest that the antioxidant capacity of hMSCs was weakened by SIRT3 silencing via inhibition of the expression and activity of MnSOD and CAT via transferring FoxO3a out of the nucleus.

### 3.4 Infarct size decreases after transplantation of SIRT3‐overexpressed O‐hMSCs

**Figure 6:**
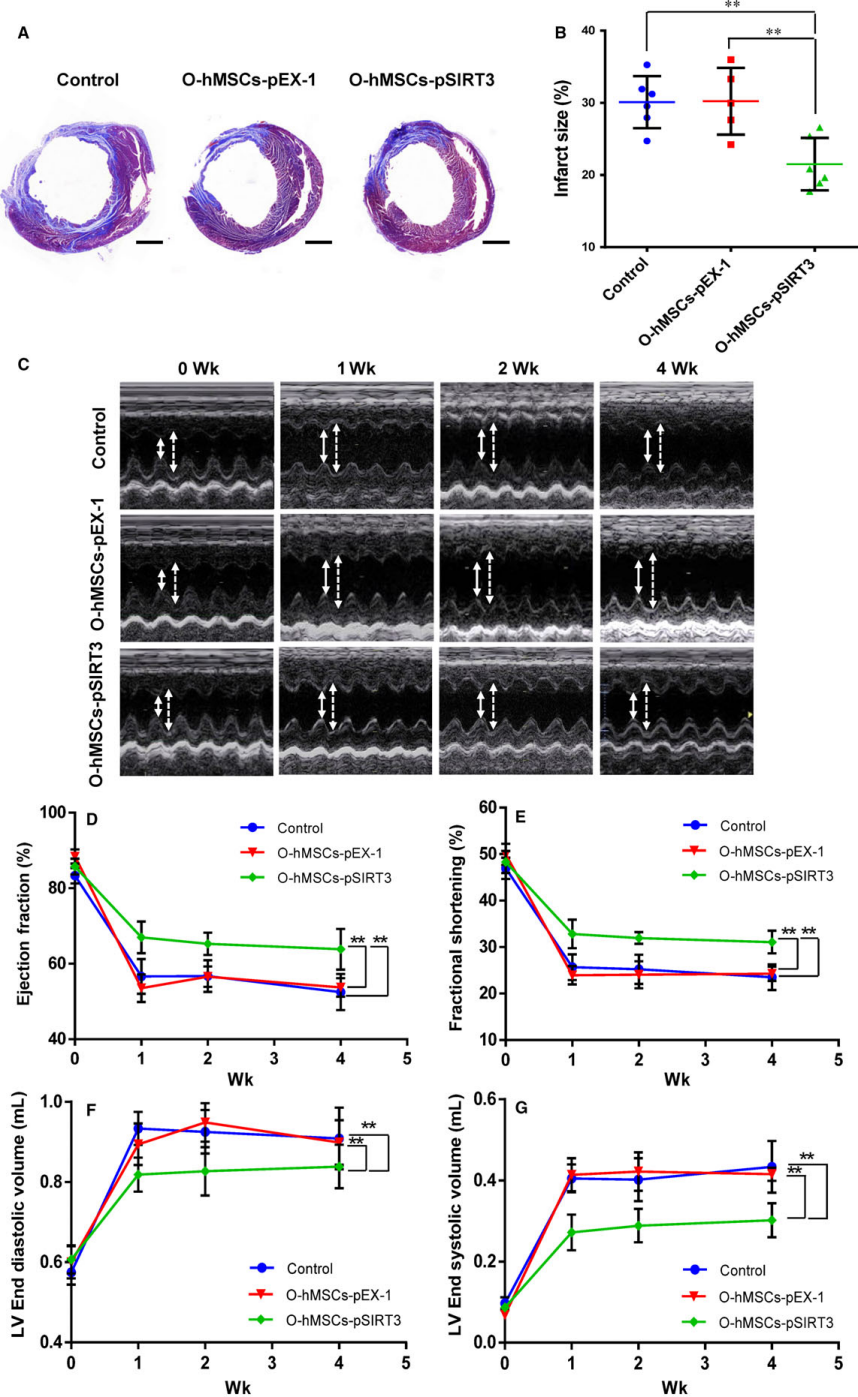
Infarct size was decreased and cardiac function was enhanced after cell transplantation with old human mesenchymal stem cells (hMSCs) modified by sirtuin3 (SIRT3). A, Masson's Trichrome staining of the infarct size 4 wk after cell transplantation in the three groups (blue = collagen; red = myocardium). Scale bars represent 2 mm. B, The infarct size of the O‐hMSCs‐pSIRT3 group was significantly smaller than the medium control and O‐hMSCs‐pEX‐1 groups 4 wk after myocardial infarction (MI), and there was no significant difference between the medium control and pEX‐1 groups. C, Representative echocardiography images from the three groups before and 1, 2 and 4 wk after MI (solid lines: left ventricular end‐systolic diameter; dashed lines: left ventricular end‐diastolic diameter). D,E, The ejection fraction (EF) and fractional shortening (FS) of the O‐hMSCs‐pSIRT3 group after MI were significantly higher than the medium control and O‐hMSCs‐pEX‐1 groups, and there was no significant difference between the medium control and O‐hMSCs‐pEX‐1 groups. F,G, The left ventricular end diastolic volume (LVEDV) and left ventricular end systolic volume (LVESV) of the O‐hMSCs‐pSIRT3 group after MI were significantly smaller than that of the medium control and O‐hMSCs‐pEX‐1 groups, and there were no significant differences between the medium control and O‐hMSCs‐pEX‐1 groups. (ANOVA; **P < 0.01; n = 6/group)

Four weeks after MI and cell transplantation, computerized morphometric analysis demonstrated a significantly smaller infarct size in the O‐hMSCs‐pSIRT3 group than the medium control and O‐hMSCs‐pEX‐1 groups, and there were no significant differences between the medium control and O‐hMSCs‐pEX‐1 groups (Figure 6A,B, n = 6/group). These results suggest that transplantation of SIRT3‐modified O‐hMSCs decreases myocardial infarct size and reverses adverse ventricular reconstruction after MI.

### 3.5 Cardiac function is enhanced following transplantation of SIRT3‐overexpressed O‐hMSCs

Echocardiography was conducted before and 1, 2 and 4 weeks after MI. Ejection fraction (EF) and fractional shortening (FS) significantly increased in the O‐hMSCs‐pSIRT3 group compared to the medium control and O‐hMSCs‐pEX‐1 groups, and there were no significant differences between the medium control and O‐hMSCs‐pEX‐1 groups (Figure 6C‐E, n = 6/group). The LVEDV and LVESV of the O‐hMSCs‐pSIRT3 group after MI were significantly smaller than that of the medium control and O‐hMSCs‐pEX‐1 groups, and there were no significant differences between the medium control and O‐hMSCs‐pEX‐1 groups (Figure 6F,G, n = 6/group). These results suggest that transplantation of SIRT3‐modified O‐hMSC improves cardiac function after MI.

### 3.6 SIRT3 overexpression decreases number of apoptotic cells and increases number of remaining transplanted cells

**Figure 7:**
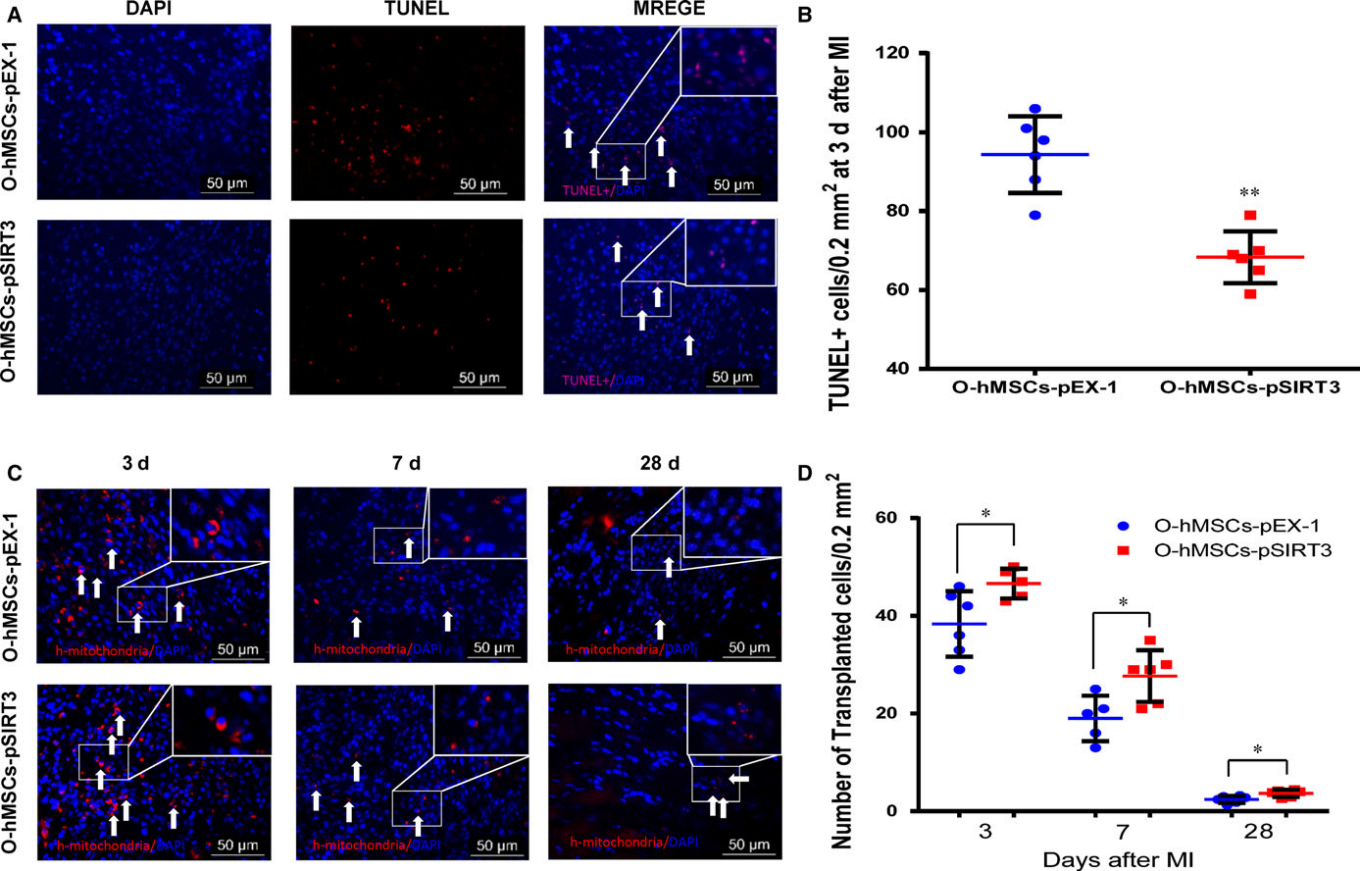
The number of apoptotic cells decreased and the number of surviving old human mesenchymal stem cells (hMSCs) increased after cell transplantation with sirtuin3 (SIRT3)‐overexpressing old hMSCs. A, Terminal dUTP nick‐end labelling (TUNEL) staining of apoptotic cells (arrows) in the two groups. Scale bars in (A,C) represent 50 μm. B, The number of apoptotic cells was significantly less in the O‐hMSCs‐pSIRT3 than the O‐hMSCs‐pEX‐1 group 3 d after cell transplantation. C, Surviving O‐hMSCs (arrows) in the two groups. D, The number of O‐hMSCs that survived was significantly greater in the O‐hMSCs‐pSIRT3 than the O‐hMSCs‐pEX‐1 group 3, 7 and 28 d after cell transplantation. (ANOVA; *P < 0.05; **P < 0.01; n = 6/group)

To further investigate the effect of SIRT3 on the function of O‐hMSCs, SIRT3‐modified O‐hMSCs were transplanted into the local hypoxic‐ischaemic region after MI in vivo. The number of apoptotic cells (TUNEL+) in the border region was significantly less in the O‐hMSCs‐pSIRT3 group than O‐hMSCs‐pEX‐1 group 3 days after cell transplantation (Figure 7A‐B, n = 6/group). To investigate the survival of O‐hMSCs in the local hypoxic‐ischaemic region, immunofluorescence staining of human‐specific mitochondria was used. The number of O‐hMSCs in the infarct border zone was significantly greater in the O‐hMSCs‐pSIRT3 group than the O‐hMSCs‐pEX‐1 group 3, 7 and 28 days after cell transplantation (Figure 7C‐D, n = 6/group). These results suggest that the apoptosis and survival of O‐hMSCs was optimized by SIRT3 overexpression.

## 4 DISCUSSION

The efficacy of current therapies for ischaemic heart diseases in aged patients is limited.^2^, ^3^ In previous pre‐clinical studies, young MSC transplantation has been demonstrated as an effective therapy for ischaemic heart diseases.^5^ The use of hMSCs is safe and feasible in the clinical setting, and these cells have been used in multiple clinical trials.^8^ However, most studies used autologous O‐hMSCs because most patients with ischaemic heart diseases are older.^7^, ^23^ The efficacy of these aged cells was found to be unsatisfactory potentially because of their decreased function.^9^ Improving the function of O‐hMSCs may improve the therapeutic effect of autologous stem cell transplantation in older patients.

Sirtuin3 (SIRT3) is a class III deacetylase depending on the nicotinamide adenine dinucleotide (NAD+) and belongs to the highly conserved Sirtuin family.^11^, ^12^, ^19^ As a mitochondria‐localized protein, SIRT3 mainly exists in tissues and organs that are rich in mitochondria such as the kidney, brain, heart and liver.^18^ Human SIRT3 has two isoforms: fl‐SIRT3 and sh‐SIRT3.^16^, ^17^, ^18^, ^19^ Previous studies, including our own, have shown that the short isoform of SIRT3 has the most functional significance.^16^, ^17^, ^18^, ^19^ In the current study, the expression level of sh‐SIRT3 was down‐regulated by oxidative stress which is consistent with previous research.^16^, ^17^, ^18^ SIRT3 has been shown to be protective against oxidative stress, but the majority of these studies were performed on animals, cell lines and tumour cells.^17^, ^20^, ^21^, ^24^, ^25^, ^26^ Although we have demonstrated that the antioxidant capacity of Y‐hMSCs was enhanced by SIRT3 overexpression,^10^ the function of SIRT3 in O‐hMSCs, which are more relevant to clinical applications, was unknown. Here, we investigated the effects of SIRT3 on O‐hMSCs, including its ability to protect O‐hMSCs against oxidative stress.

To evaluate the effects of SIRT3 on O‐hMSCs under oxidative stress, H_2_O_2_ treatment was used. The results indicated that cell apoptosis increased and survival decreased in control and experimental groups after H_2_O_2_ stress. Cell apoptosis decreased and survival increased in the pSIRT3‐transfected group, while cell apoptosis increased and survival decreased in the siRNA‐transfected group compared with the control group after oxidative stress. Thus, SIRT3 overexpression enhanced the antioxidant capacity of O‐hMSCs while silencing SIRT3 expression decreased antioxidant capacity.

It has been demonstrated that the expression of MnSOD and CAT can be up‐regulated by SIRT3 overexpression.^10^, ^17^ In agreement with previous studies, the expression levels of MnSOD and CAT were found to be positively associated with SIRT3 expression in the current study. The expression levels of SIRT3, MnSOD and CAT were down‐regulated by H_2_O_2_ treatment, revealing that SIRT3, MnSOD and CAT were involved in oxidative stress. The expression levels of SIRT3, MnSOD and CAT were higher in the SIRT3 group than the empty vector group, and these expression levels were decreased in the siRNA‐transfected group compared with the control group after H_2_O_2_ stress. This suggests that SIRT3 overexpression improved the antioxidant capacity of hMSCs by increasing the expression of MnSOD and CAT. The deacetylation of protein is a common phenomenon in mitochondria.^27^ As a deacetylase, the main function of SIRT3 is to deacetylate the transcription factor FoxO3a which can enter the nucleus and activate expression of MnSOD and CAT to reduce ROS content.^20^, ^21^, ^22^, ^28^ In this study, the expression level of FoxO3a in nuclear fraction was also positively associated with the expression of SIRT3, MnSOD and CAT. These results suggest that SIRT3 overexpression enhanced antioxidant capacity via the FoxO3a‐MnSOD and CAT pathway in O‐hMSCs, similar to that which occurs in other classes of cells. There are some limitations to this research. As previously reported, SIRT3 regulates activation of oxidative metabolism and mitochondrial fatty‐acid oxidation through deacetylation of numerous mitochondrial enzymes.^29^, ^30^ In the current study, we did not investigate mitochondrial function because we focused on cellular function. Future work will investigate the comprehensive function of SIRT3.

Studies have verified that the benefit of stem cell transplantation is dependent upon the number of residual transplanted cells in the local hypoxic‐ischaemic environment after MI.^31^, ^32^ We have confirmed that the ability of O‐hMSCs to resist oxidative stress in vitro was improved by SIRT3 overexpression. We further proposed that the number of remaining stem cells would be increased after O‐hMSC transplantation and that the therapeutic effect of cell transplantation would be improved by SIRT3 overexpression in vivo.

In the in vivo experiment, the number of apoptotic cells was found to be less in the O‐hMSCs‐pSIRT3 group than the O‐hMSCs‐pEX‐1 group after short‐term acute ischaemia and hypoxia (3 days after MI). Furthermore, O‐hMSC survival decreased with time after MI. However, more O‐hMSCs survived after pSIRT3 transfection than pEX‐1 transfection in acute and chronic ischaemia and hypoxia. These results suggest that SIRT3 protects O‐hMSCs against anoxia and ischaemic injury in vivo and the protective effect is persistent. Although we found that ejection fraction and fractional shortening significantly declined after MI, the reduction in cardiac function was relatively less in the O‐hMSCs‐pSIRT3 group than the O‐hMSCs‐pEX‐1 group 7 days after MI. Twenty‐eight days after MI, cardiac function remained at a higher level in the O‐hMSCs‐pSIRT3 group than the control group. LVEDV and LVESV significantly increased after MI. However, the increment in volume was relatively smaller in the O‐hMSCs‐pSIRT3 group than the control groups, especially for LVESV. We further discovered that infarct size was the smallest in the O‐hMSCs‐pSIRT3 group 28 days after MI, indicating that implanting O‐hMSCs that overexpressed SIRT3 significantly alleviated the ventricular remodelling that occurs during the long‐term recovery from MI. We thus demonstrated the protective effect of SIRT3 on O‐hMSCs. SIRT3 overexpression may represent a means by which to improve the therapeutic effect of O‐hMSC transplantation for the treatment of ischaemic heart diseases.

## 5 CONCLUSION

**Figure 8:**
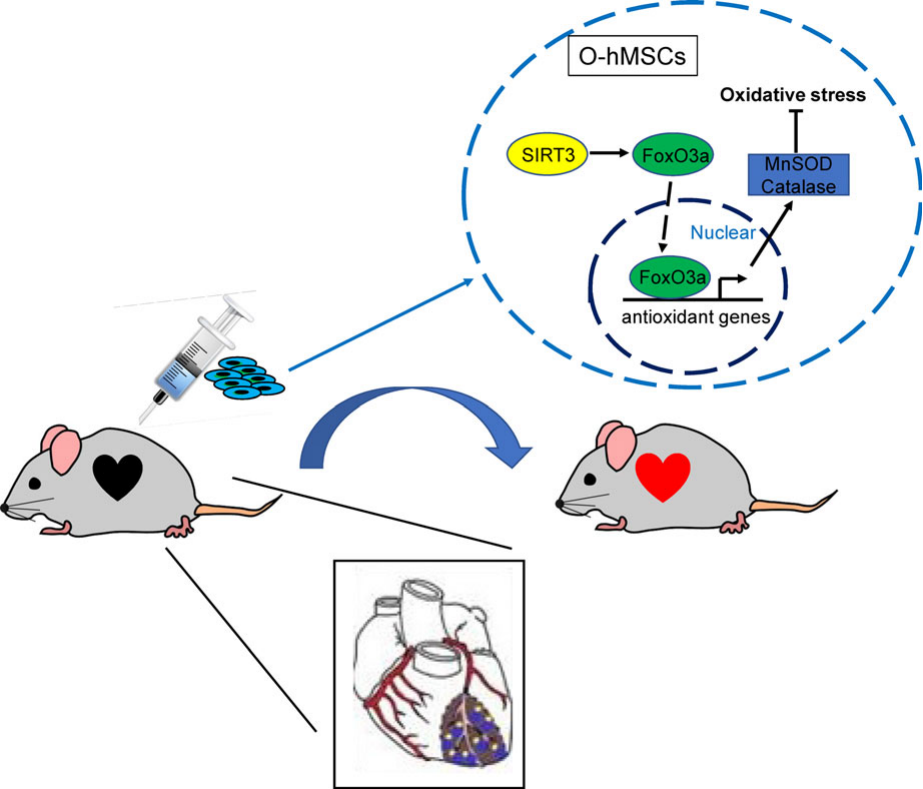
Summary of findings. Sirtuin3 (SIRT3) protects old human mesenchymal stem cells (O‐hMSCs) against oxidative stress by positively regulating Mn‐superoxide dismutase (MnSOD) and catalase via increasing the expression of forkhead box O3a in the nucleus. The efficacy of cell therapy was enhanced after transplantation with SIRT3‐overexpressing O‐hMSCs after MI

Sirtuin3 protects aged hMSCs against oxidative stress by positively regulating antioxidant enzymes including MnSOD and CAT via increasing the expression of FoxO3a in the nucleus (Figure 8). In vivo*,* the number of apoptotic cells was reduced and the number of transplanted cells that survived was increased by SIRT3 overexpression in O‐hMSCs. Cardiac function was enhanced, and infarct size was decreased after transplantation with SIRT3‐overexpressing O‐hMSCs after MI (Figure 8).

## CONFLICT OF INTEREST STATEMENT

The authors confirm that there are no conflicts of interest.

## AUTHORS’ CONTRIBUTIONS

D‐YZ, C‐FZ, B‐CF and X‐QW performed the research; LS participated in data processing and statistical analyses; WC, WL, K‐YL, C‐YM and S‐LJ provided experimental technical support; G‐QD performed the echocardiography; D‐YZ and HT wrote the manuscript; and R‐KL, HT and LS jointly conceived the study, and drafted the manuscript.
